# Evolutionary profiles and complex admixture landscape in East Asia: New insights from modern and ancient Y chromosome variation perspectives

**DOI:** 10.1016/j.heliyon.2024.e30067

**Published:** 2024-04-30

**Authors:** Zhiyong Wang, Mengge Wang, Liping Hu, Guanglin He, Shengjie Nie

**Affiliations:** a School of Forensic Medicine, Kunming Medical University, Kunming, 650500, China; b Institute of Rare Diseases, West China Hospital of Sichuan University, Sichuan University, Chengdu, 610000, China; c Center for Archaeological Science, Sichuan University, Chengdu, 610000, China; d Faculty of Forensic Medicine, Zhongshan School of Medicine, Sun Yat-sen University, Guangzhou, 510275, China

**Keywords:** Founding lineage, Y-chromosome genetic diversity, Genomic resources, East Asian, Evolutionary history, Ancient DNA

## Abstract

Human Y-chromosomes are characterized by nonrecombination and uniparental inheritance, carrying traces of human history evolution and admixture. Large-scale population-specific genomic sources based on advanced sequencing technologies have revolutionized our understanding of human Y chromosome diversity and its anthropological and forensic applications. Here, we reviewed and meta-analyzed the Y chromosome genetic diversity of modern and ancient people from China and summarized the patterns of founding lineages of spatiotemporally different populations associated with their origin, expansion, and admixture. We emphasized the strong association between our identified founding lineages and language-related human dispersal events correlated with the Sino-Tibetan, Altaic, and southern Chinese multiple-language families related to the Hmong-Mien, Tai-Kadai, Austronesian, and Austro-Asiatic languages. We subsequently summarize the recent advances in translational applications in forensic and anthropological science, including paternal biogeographical ancestry inference (PBGAI), surname investigation, and paternal history reconstruction. Whole-Y sequencing or high-resolution panels with high coverage of terminal Y chromosome lineages are essential for capturing the genomic diversity of ethnolinguistically diverse East Asians. Generally, we emphasized the importance of including more ethnolinguistically diverse, underrepresented modern and spatiotemporally different ancient East Asians in human genetic research for a comprehensive understanding of the paternal genetic landscape of East Asians with a detailed time series and for the reconstruction of a reference database in the PBGAI, even including new technology innovations of Telomere-to-Telomere (T2T) for new genetic variation discovery.

## 1 Introduction

East Asia, which geographically consists of China, Mongolia, the Korean Peninsula, and the Japanese archipelago, harbors more than 200 languages in seven linguistic families (Altaic, Austro-Asiatic (AA), Austronesian (AN), Tai-Kadai (TK), Hmong-Mien (HM), Sino-Tibetan (ST) and Indo-European), along with numerous other ethnic groups. Due to complex patterns of geographical, linguistic, and ethnic composition, the region has tremendous human genetic and cultural diversity [1]. Previous genetic studies have attempted to elucidate the formative processes underlying observed patterns of genetic diversity and the corresponding influencing factors using three types of genetic materials, including maternally inherited mitochondrial DNA (mtDNA), paternally inherited male-specific region of the Y chromosome (MSY) and biparentally inherited genome-wide autosomal DNA [[2], [3], [4], [5]]. In contrast to autosomal DNA, mtDNA and MSY exhibit haploid inheritance, which can escape reshuffling effects, and their polymorphisms, formed by orderly cumulative variations, serve as faithful records of human origin, migration, and expansion [6,7]. The effective population size of MSY is one-quarter that of autosomes, making it susceptible to genetic drift and quickly resulting in genetic variations with population and geographic specificity [8]. Moreover, the development of agriculture since the Neolithic shed light on the emergence of patriarchal societies in East Asia [[9], [10], [11]]. The paternally inherited MSY holds an advantage in identifying and documenting the historical demographic processes of dominant populations. Hence, MSY remains an essential object for studying human evolution because of the lack of genome-wide data from modern and ancient populations in early studies.

In the past two decades, there have been two phases in the study of human evolution based on two Y-chromosomal genetic markers—transforming from independently applied short tandem repeats (STRs) and single nucleotide polymorphisms (SNPs)—to the combination of these two genetic markers with next-generation sequencing (NGS)-based technologies. The first phase roughly characterized modern humans' patterns of origin and migration through genotyping several or dozens of Y-chromosomal STRs (Y-STRs) or Y-chromosomal SNPs (Y-SNPs) [[12], [13], [14]]. The second phase benefited from the use of next-generation sequencing (NGS) technologies, mainly hybridization capture using probes, which allowed us to precisely capture more than 10 Mb of the Y chromosome despite its highly repetitive sequences. As a result, this approach deepened the resolution of the paternal lineage and provided more phylogenetic information about human genetic history [[15], [16], [17]].

A wealth of knowledge on the phylogeography of the Y-chromosomal haplogroups and the paternal founding lineages of ethnolinguistically diverse groups provides the basis for pedigree search and paternal biogeographical ancestry inference (PBGAI) in forensic investigations based on the above research on human evolution [18]. However, before applying the current state-of-the-art Y-chromosome phylogeny to pedigree searches and PBGAI, it is essential to distinguish whether the Y-chromosome haplogroup diversity is clustered or clined [2]. In the second phase of genetic studies of the Y chromosome, numerous high-resolution Y-SNP panels have been developed, and fine-scale paternal population structures have been illuminated based on spatiotemporally different Y chromosome genomes. The admixture process of East Asian ethnic groups involves multiple levels and multiple waves. Consequently, the Y-chromosome genetic profile is a cline in the geographic distribution of frequencies of major haplogroups and clusters in a high-resolution phylogenetic tree [19,20].

**Fig. 1:**
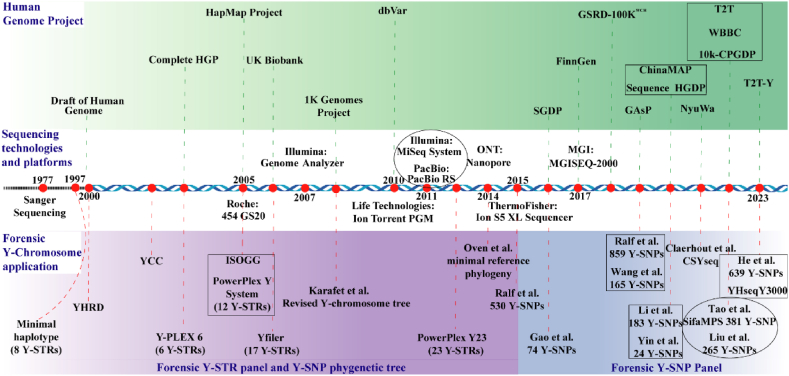
**The timeline of different human genome projects, sequencing techniques, platforms and forensic applications.** Since our focus is on developing forensic Y-SNP panels, the other events are only partially presented. The abbreviations are presented as follows. **HGP**: Human Genome Project. **Hapmap**: Haplotype Map. **SGDP**: Simons Genome Diversity Project. **GSRD-100K**^**WCH**^: Genome Sequence of Rare Disease-100k^West China^. **GAsP**: GenomeAsia 100K Project. **ChinaMAP**: China Metabolic Analytics Project. **HGDP**: Human Genome Diversity Project. **T2T**: Telomere-to-Telomere. **WBBC**: Westlake BioBank for Chinese. **10K-CPGDP**: 10K Chinese Person Genomic Diversity Project.

**Fig. 2:**
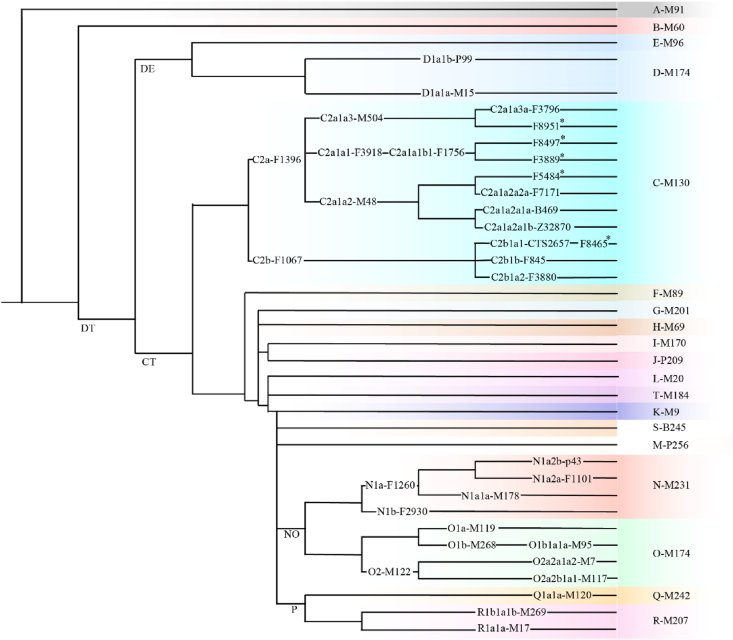
**The simplified Y-chromosome phylogenetic tree.** The haplogroups that appear in the review are shown in the tree. The topology structure and nomenclature are compatible with the International Society of Genetic Genealogy (ISOGG) Y-DNA Haplogroup Tree 2019–2020 version 15.73 (http://www.isogg.org/tree/). * indicates that the haplogroup was present in previous literature but was not included in the ISOGG.

Scientists in the field of forensic science have developed several high-resolution Y-chromosome genotyping systems with lineage-informative Y-SNPs for pedigree search and PBGAI, aiming to improve the resolution from continents to a more detailed geographic region or population by investigating Y-chromosome genetic profiles across different geographic regions or ethnic groups [[21], [22], [23], [24], [25], [26], [27], [28]] (Fig. 1). Therefore, this review first presents a general landscape of Y chromosome diversity among Paleolithic-to-historic and modern ethnolinguistically diverse East Asians. Then, we elucidated the key factors contributing to the observed patterns of genetic diversity. Finally, we summarized the significant applications of Y-chromosome genetic diversity in forensic science, the anthropological field, and others and presented an outlook on PBGAI in forensic practice, focusing on the potential impact of third-generation sequencing in the future. In addition, due to the existence of different categories of phylogenetic trees and haplogroup nomenclature [29], we used the International Society of Genetic Genealogy (ISOGG) Y-DNA Haplogroup Tree 2019–2020 version 15.73 (http://www.isogg.org/tree/) in this review, and the haplogroups not included in ISOGG 2019–2020 were named with their upstream haplogroups (Fig. 2).

## 2 The paternal genetic architecture in pre-Neolithic East Asia

Genetic evidence generally supported the “Out of Africa” hypothesis that modern humans originated in Africa and migrated to Eurasia approximately 60K years ago (kya) [30]. However, modern humans were not always alone in migrating out of Africa. They occasionally encountered and interbred with now-extinct archaic humans, specifically Denisovans and Neanderthals. Advancements in ancient DNA research have allowed us to determine the extent and frequency of genetic exchange between modern and archaic humans at the level of autosome DNA [[31], [32], [33]]. However, there is a notable discrepancy in phylogenetic affiliation between monophyletic genetic markers and autosomal DNA, in which late Neanderthals appear to be more closely related to modern humans than Denisovans [34,35]. This suggests that the monophyletic genetic markers of late Neanderthals have been replaced by a lineage related to modern humans. The Ust’-Ishim from western Siberia, dated 45,000 years ago, represents one of the earliest anatomically modern humans known to date. This individual carries the deep-rooted mutation M2308, which defines the haplogroup NO on the Y chromosome [36]. The majority of the extant East Asian paternal lineage belonged to the downstream haplogroup NO, which indicated a degree of genetic continuity and persistence in Eurasia [[37], [38], [39], [40]].

Molecular anthropologists initially unveiled the time and route of modern human arrival in East Asia through genotyping Y-STRs plus Y-SNPs. Subsequently, three postulated models were proposed, including the southern route followed by northward migration, the northern route followed by southward migration, and the independent origin of both the southern and northern routes during the Paleolithic period [37,38,41]. Modern humans who initially settled in East Asia originated from the dispersal of pre-Neolithic hunter-gatherer communities. The southern route primarily facilitated the dispersal of the Y-chromosome haplogroups O-M175, C-M130, N-M231, and D-M174 [42]. The migrants carrying these paternal lineages moved from Southeast Asia to East Asia at different times, as determined by analyzing the diversity of Y-STR haplotypes combined with Y-SNP haplogroups from various geographic regions [[43], [44], [45], [46], [47]]. The subhaplogroups of O-M175 might enter East Asia via two routes. The Y-chromosome haplogroups O2a2a1a2-M7 and O2a2b1a1-M117 likely diffuse via the western route from Myanmar to East Asia, while the Y-chromosome haplogroups O1a-M119 and O1b1a1a-M95 might spread via the eastern coastal route, passing through Hainan Island [48,49]. The northern route, initiated during postglacial migration (15-18kya), likely coincided with the spread of the Y chromosome haplogroups Q-M242 and R-M207 [38]. The general composition of major Y-chromosome haplogroups in East Asia was formed during the pre-Neolithic period.

## 3 Dynamic demography of geographically different East Asians since the Neolithic era

**Fig. 3:**
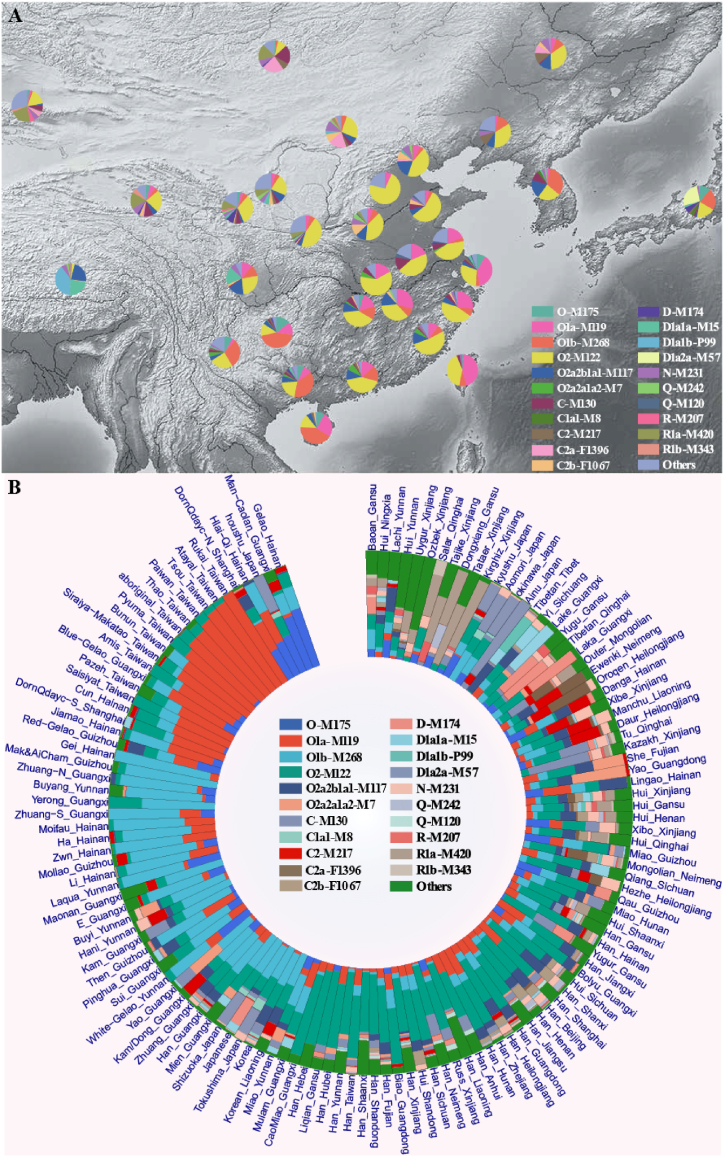
**Distribution patterns of Y chromosome haplogroups in modern populations.** (A) The phylogeography of haplogroups in East Asia. In China, we calculate haplogroup frequency by province. (B) The distribution patterns of haplogroups in different ethnolinguistic populations. The detailed information is presented in Table S1.

Previous genome-wide studies of ancient DNA have revealed more profound differences in the genetic structure of various East Asians during the pre-Neolithic period than during the present day, which implies extensive admixture since the Neolithic era [50,51]. The innovation and development of agriculture in the Yellow and Yangtze River Basins significantly propelled the expansion and migration of ancient East Asians during the Neolithic [[50], [51], [52]]. The northern millet-related populations profoundly contributed to the gene pool of the proto-ST populations, while the southern rice-related populations significantly influenced the genetic landscape of southern Chinese multilanguage families. Additionally, bidirectional gene flow between these two agriculturally related populations has emerged and continues to increase. The intensive migration of nomadic-related populations within the Eurasia steppe is a central theme of the dynamic history of the Bronze and Iron Age in East Asia, primarily due to the development of metallurgy technologies [50,[53], [54], [55]]. The admixture events resulting from nomadic-related populations had varying degrees of impact on East Asia's northern and northwestern regions. Meanwhile, the formation of states and civilizations in the central regions of East Asia promoted migration among East Asians. Historically, the migration of East Asians has been driven mainly by regime change, warfare, and famine, resulting in three major north-to-south migrations [56,57]. Therefore, the significant population expansion and extensive admixture since the Neolithic era suggest that the composition and frequency of Y-chromosome haplogroups in present-day East Asians are complex and multifaceted (Fig. 3A and B).

In brief, four major haplogroups gradually emerged, namely, O-M175, C-M130, N-M231, and D-M174, collectively accounting for approximately 93 % of the Y chromosome in East Asia. Other haplogroups, such as E-SRY4064, G-M201, H-M69, I-M170, J-P209, L-M20, Q-M242, R-M207, and T-M70, made up approximately 7 % of the total population [38]. While these haplogroups did not present strict geographical or ethnolinguistic distribution patterns in East Asia, it is important to note that certain subhaplogroups were distributed at high frequencies in specific geographic regions or particular ethnolinguistic groups (Fig. 3A and B). In addition, studying ancient Y chromosomes can provide a distinct insight into male-related population migrations or replacements by observing the dynamic variations in the composition or frequency of Y chromosome haplogroups over time compared to complex modeling of autosomal DNA [[58], [59], [60]]. Consequently, we collected paternal haplogroup results from published ancient DNA and corresponding bam files spanning different periods to dissect the dynamic genetic history of East Asia. We used pathPhynder software specifically designed for ancient DNA to verify the Y chromosome haplogroup results [61].

The genetic scenarios of paternal lineages and genome-wide diversity in East Asia are strongly connected with the language family [47,48,[62], [63], [64]]. Therefore, this review will concentrate on the ST, Altaic, HM, AA, TK, and AN strains to determine paternal genetic diversity in East Asia. Here, we will illustrate the origin of founding paternal lineages specific to a particular ethnolinguistic group. Typically, paternal lineages are derived from population admixtures. Furthermore, genetic diversity is inherently derived from mutation, genetic admixture, genetic drift, and natural selection. The patterns of paternal genetic diversity are often influenced by cultural practices, geography, historical events, and so on. Therefore, we will also consider the impact of these factors on genetic diversity in East Asia.

### 3.1 Origin and expansion of the ST-speaking populations

**Fig. 4:**
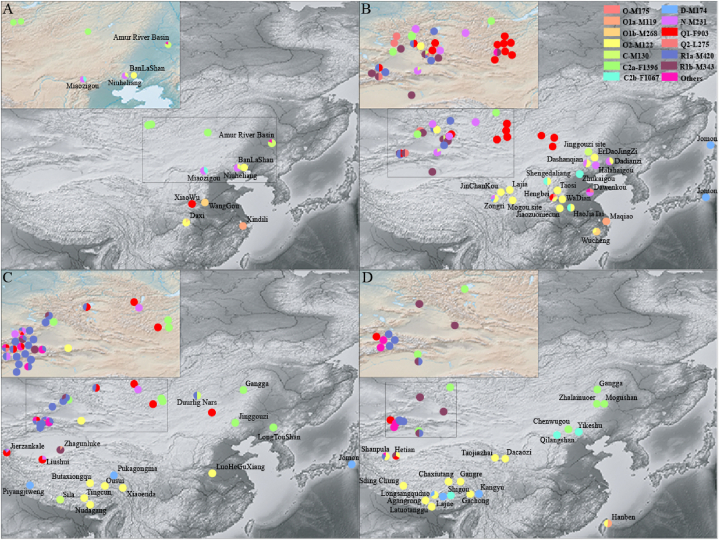
**Distribution patterns of East Asian-dominant haplogroups in spatiotemporally different populations inferred from ancient DNA over time.** (A) Patterns of the Y chromosome lineages inferred from ancient DNA extracted 5300 years ago. (B) Patterns of the Y chromosome lineages inferred from ancient DNA ranging from 5300 to 3000 years BP. (C) Patterns of the Y chromosome lineages inferred from ancient DNA from 3000–2000 years BP. (D) Patterns of the Y chromosome lineages inferred from ancient DNA from 2000 years BP. Due to the overlap of the Xinjiang and Mongolia sites, we show the overlap of the sites in the upper left corner of each figure. The detailed information is shown in Table S2.

The ST language family generally consists of Sinitic and TB, which are collectively spoken by approximately 1.5 billion native speakers. Evidence from the linguistic phylogeny, archaeology, and ancient DNA supported the northern-origin hypothesis, suggesting that the ancient populations speaking the proto-ST family and cultivating millet in the middle and upper Yellow River separated into two groups approximately 4–6 kya [51,[65], [66], [67], [68], [69]]. One group moved east and south of East Asia, spreading the Sinitic language, while the other group migrated to southwest East Asia, disseminating the TB language. Yan et al. identified three Neolithic supergrandfather lineages, namely, Oα-F11, Oβ-F46, and Oγ-M117 (subgroups of O2-M122), through the construction of a maximum parsimony phylogenetic tree using 3.9 Mb of MSY sequence data obtained from 78 East Asian Y chromosomes. These three identified lineages were widespread in East Asians and represented more than 40 % of the Han Chinese population. In addition, star-like expansions of the three lineages occurred during the Neolithic period at approximately six kya, which aligns with the northern-origin hypothesis of ST-speaking populations [15]. The ancient Y chromosomes discovered in the Yellow River and Tibetan Plateau predominantly belong to haplogroup O2-M122, accounting for the major paternal gene pool of extant Sino-Tibetan-speaking populations in East Asia, further supporting this hypothesis (Fig. 4A–D).

#### 3.1.1 Sinitic-speaking populations

In these two groups, Sinitic-speaking Han Chinese occupied approximately 91 % of the present-day East Asians. According to the historical record, the Han Chinese are generally regarded as descendants of the ancient Huaxia tribe of northern China during the Neolithic period, and the appearance of “Han Chinese” is generally thought to have occurred during the Han Dynasty (206 BCE – 220 CE) [70]. O2-M122 is the dominant haplogroup in the Han Chinese [43]. As shown in Fig. 3A and B, O2-M122 is widespread in different geographic and ethnolinguistic groups, especially in Han Chinese, with extremely high frequency. Consequently, the three founding paternal lineages that emerged during the Neolithic period underwent dramatic spread and interaction in the following periods. By systematically comparing the genetic profiles of the Y chromosome and mtDNA among northern and southern Han Chinese populations, Wen et al. identified the demic diffusion model of Han culture from north to south, which is consistent with the three extensive migrations that have been documented in historical records over the past 2000 years [71]. Therefore, the proto-Han Chinese population, as the major ethnic group in present-day East Asia, has contributed to the gene pool of other ethnolinguistic groups in the region, as evidenced by the widespread distribution of haplogroup O-M122 across East Asians (Fig. 3A and B).

However, the gene flow between Han Chinese and other ethnic groups is bidirectional, and other major haplogroups also occurred in Han Chinese, such as Q-M242, N-M231, and C-M130 (Fig. 3B), but with low frequency, which might imply deep admixture since the Neolithic. Here, we will mainly concentrate on Q-M242 and N-M231. Haplogroup Q-M242 has expanded from Siberia since the end of the Last Glacial Maximum (LGM) and is now widely distributed in Eurasia and North America, and the subhaplogroups of Q-M242 have been substantially studied [[72], [73], [74], [75]]. The distribution pattern of haplogroup Q1a1a-M120 in present-day East Asians has received attention as one of the founding paternal lineages of Han Chinese. Q1a1a-M120 was found to occur widely in Mongolia at approximately three kya and presented a high frequency at the 3000-year-old Hengbei site as well as at the 2500-year-old PengYang site, which suggested that the haplogroup Q1a1a-M120 might have been integrated into the Han Chinese population from northwest East Asia to the central plain approximately 3000 years ago [51,76,77]. Interestingly, the haplogroup Q1a1a-M120 that appeared at the XiaoWu site in YangShao culture was dated to ∼7kya, which might imply that Q1a1a-M120 was involved in the formation of Han Chinese at an earlier time [78]. Wei et al. reconstructed a highly revised phylogenetic tree of haplogroup Q1a1a-M120 to subtly reveal the origin and diffusion of this haplogroup. This work demonstrated that the initial expansion of haplogroup Q1a1a-M120 occurred between 6.8kya and 5kya, approaching the earlier advance of Q1a1a-M120 in YangShao culture, followed by expansion until merging into Han Chinese at 3kya, consistent with the evidence of ancient DNA [79]. N-M231, the sister haplogroup of O-M175, underwent a counter-clockwise migration from Southeast Asia toward Europe, presenting a unique distribution that cut across language families [46,80]. In East Asia, there is a region-specific distribution of the sister haplogroups, with N1a-F1260 distributed mainly in northern East Asia and N1b-F2930 in southern East Asia [81]. However, the detailed origin and expansion of N-M231 in East Asia remain ambiguous compared to those in northern and eastern Europe and Siberia [80,82]. Ancient DNA also presented a fresh perspective that N-M231x (M128, TAT) was the predominant haplogroup of the West Liao River Valley, which was considered a cradle of Chinese civilization during the Neolithic. The dynamic change in this region occurred mainly in the Bronze Age, with a decrease in N-M231x (M128, TAT) and an increase in O2a2-M117 and N1a1-TAT, which might have been influenced by nomadic groups of Eastern Eurasian Steppe and Yellow River millet farmers [83]. Recent research revealed that N1a2a-F1101, the sister lineage of N1a2b-P43 that showed much greater affinity for the Uralic-speaking populations, contributed to the gene pool of Han Chinese in the Bronze Age, playing essential roles in the formation of the early state and civilization, consistent with the evidence of ancient DNA of Xiguan cemetery where the haplogroup N1a2a-F1101 was first detected in China [84,85]. N1b-F2930 accounts for a certain proportion of present-day Han Chinese, but its origin in Han Chinese is unclear. At the same time, the lack of subdivision of N-M231 in the West Liao River Valley makes its origin and diffusion elusive in China, and more ancient DNA is expected to provide detailed clues about the origin of N-M231, except for N1a2a-F1101. The appearance of haplogroup C-M231 (mostly C2b-F1067) in Han Chinese will be explained following this passage. Overall, the populations classified into haplogroups N1a2a-F1101 and Q1a1a-M120 integrated into the Han Chinese population at least in the Bronze Age and contributed substantially to the formation of state and early civilizations.

#### 3.1.2 TB-speaking populations

TB-speaking populations are predominantly found in the southwestern region of East Asia, encompassing the Tibetan Plateau, Yunnan-Guizhou Plateau, and surrounding areas. The evidence derived from the Y chromosome elucidates that the contemporary genetic makeup of TB-speaking populations can be traced to the predominant admixture of two groups in the Neolithic period. The hunter-gatherer populations initially settled on the Tibetan Plateau in the Paleolithic period with haplogroup D-M174 and ancient northern East Asians who migrated westward from the upper-middle Yellow River basin with haplogroups O2a2b1-M134 (xM117) and O2a2b1a1-M117 [12,69,[86], [87], [88]]. Further research showed that there might have been another Neolithic expansion of TB-speaking populations. The ancient groups from the Yellow River basin directly moved to the southwest. Eventually, they arrived in southern East Asia with haplogroup Oα1c1a-Z25929 (downstream of O2a2b1a1-M117) and no or a minor proportion of D-M174, as revealed by a high-resolution phylogeny of the paternal lineages of TB-speaking populations [89]. First, O2a2b1a1-M117 is the most prevalent haplogroup in TB-speaking populations because of the influence of ancient populations from the upper-middle Yellow River basin. Second, two dominant subhaplogroups of D-M174 (D1a1a-M15 and D1a1b-P99) present different distribution landscapes in TB-speaking populations. D1a1b-P99 is generally restricted to TB-speaking populations and is particularly predominant in Tibetan and Qiangic populations. Surprisingly, a previous study revealed that D1a1b-P99 accounted for up to 70.21 % of the Qiangic-speaking Pumi population [47]. However, D1a1a-M15 is widely distributed in East Asia and appears not only in TB-speaking populations but also in TK- and HM-speaking populations [47]. A recent ancient DNA study focused on Tibetan Plateau populations showed that haplogroup D-M174 first appeared at the Zongri site, dated to 4500 years ago, suggesting that haplogroup D-M174 contributed to the gene pool of TB-speaking populations at least 4500 years ago [90]. However, the absence of downstream haplogroups of D-M174 hindered the dissection of the paternal genetic history of deeply divergent lineages (D1a1a-M15 and D1a1b-P99) in TB-speaking populations. Moreover, TB-speaking populations have a certain percentage of haplogroup N-M231. In particular, the Yi population exhibits a relatively high frequency of N-M231 [46]. Furthermore, a recent genetic investigation revealed that most N-M231 within this population belonged to the N1b-F2930 subclade [91]. Additionally, haplogroup F-M89 (xGHIJK), a rare deep-root lineage [92], has been exclusively identified in East Asia and Southeast Asia, with relatively high frequencies observed in the Lahu population from Yunnan Province and the Yi population from Sichuan Province [91,93]. This phenomenon of the identified population-specific lineages might indicate that East/Southeast Asia is the dispersal center of all surviving non-African Y chromosome lineages [94]. However, this conclusion requires further clarification through additional DNA sequences from modern and ancient populations and multidisciplinary evidence.

In summary, the Neolithic expansion and migration of ST populations obeyed the farming-and-language-dispersal hypothesis that the expansion of populations and languages was agriculture-derived [95] and that the north-to-south migration of Han Chinese in the historical period was mainly driven by warfare and famine in line with historical records. The change in subsistence strategy between agriculture and pastoralism resulting from climate change may influence the migration and diffusion of paternal lineages, such as Q1a1a-M120 and N1a2a-F1101.

### 3.2 Origin and dispersal history of the Altaic-speaking populations at the crossroads of Siberia and East Asia

Northern East Asia harbors a complex demographic history, and the expansion of nomadic populations and regime changes in the Eurasian Steppe powerfully shape the landscape of paternal lineages. The Altaic language family can generally be divided into Tungusic, Mongolic, and Turkic groups. East Asian populations who speak these languages are mainly distributed in the northern regions with a distinct paternal lineage composition. First, C2-M231 is the dominant haplogroup in Altaic-speaking populations, but there is a stratification of the sublineage composition of C2-M231 within the Altaic-speaking populations distributed in different regions of northern East Asia [44,96]. In general, haplogroup C2-M231 can be divided into two subhaplogroups, C2a-F1396 and C2b-F1067, and the distribution pattern of these two subhaplogroups is region-specific, where C2a-F1396 has a more northern distribution than C2b-F1067 in present-day populations [15,[97], [98], [99]]. Haplogroups C2a-F1396 include three predominant subhaplogroups, C2a1a3-M504, C2a1a1-F3918, and C2a1a2-M48, which are widely distributed in distinct subgroups of Altaic-speaking populations. Zerjal et al. reported that the Y-chromosome lineage C3*-Star Cluster (previously named) accounted for 8 % of the males living in a large part of Asia, revealing the western expansion of the Mongol Empire [100]. Subsequently, as all samples of the C3*-Star Cluster were derived from F3796, Wei et al. redefined this paternal lineage as C2a1a3a-F3796 and found that C2a1a3a-F3796 was one of the founding paternal lineages of Mongolic-speaking populations according to a revised phylogenetic tree of the C3*-Star Cluster. C2a*-F8951 (not included in ISOGG 2019), the sister haplogroup of C2a1a3a-F3796, is mainly found in Daur and Manchu populations and is related to the founding paternal lineage of the Aisin Gioro family [20,98,101]. Significantly, haplogroups C2a1a3a-F3796 and C2b-F8951 exhibited region-specific distributions. C2a1a3a-F3796 are mainly distributed in the western part of the Greater Khingan Mountains, while C2b-F8951 are mainly distributed in the eastern part of the Greater Khingan Mountains. A previous study revealed that C3*-DYS448del (null in Y-STR DYS448 loci) was derived from C2a1a1b1-F1756, downstream of C2a1a1-F3918, and was an essential paternal lineage in Altaic-speaking populations [97]. The split of the two lineages of C2a1a1b1-F1756 is related to west‒east divergence; C2a*-F3889 (not included in ISOGG 2019) is more common in the eastern region of the Mongolian Plateau, while C2a*-F8497 (not included in ISOGG 2019) is more common in the western region of the Mongolian Plateau. In addition, C2a1a1-F3918 and its sublineage C2a1a1b1a-F3830 play significant roles in the paternal genetic composition of ancient nomadic populations in northern East Asia. These lineages have been frequently detected in archaeological sites associated with the Donghu, Xianbei, Rouran, and Shiwei nomads, which successively dominated the Mongolian Steppe region [102,103]. C2a1a1b1a-F3830 has contributed to the gene pool of present-day Mongolic- and Manchu-speaking populations. C2a1a2-M48 has high frequencies in Mongolic-, Tungusic- and Turkic-speaking populations, whereas the sublineage C2a*-F5484 (not included in ISOGG 2019) is the founding paternal lineage of the Tungusic-speaking populations [64]. Interestingly, there was a region-specific distribution of two sister sublineages derived from C2a1a2-M48: an east‒west distribution in which C2a-F5484 was mainly in the east and C2a1a2a2a-F7171 was mainly in the west, and a north‒south distribution in which C2a1a2a1b-Z32870 was more common in Tungusic-speaking populations of Siberia and C2a1a2a1a-B469 was more common in Tungusic-speaking populations in northeastern East Asia [64].

Compared to the distribution pattern of C2a-F1396 and its subclades, C2b-F1067 and its sublineages have distinct diffusion centers and distributions in East Asia. As shown in the ancient DNA study, the Miaozigou, Shimao, HaoJiaTai, Zhukaigou, Qilangshan, and Yikeshu sites were attributed to C2b-F1067 and had a more southerly distribution than C2a-F1396. In addition, the ancient DNA from the Shimao site showed close genetic affinity with that of Han Chinese. The appearance of C2b-F1067 at the Shimao site might suggest that C2b-F1067 contributed to the gene pool of the Han Chinese population [78,104]. C2b-F1067 has three predominant subclades, C2b1a1-CTS2657, C2b1b-F845, and C2b1a2-F3880. A previous study demonstrated that C2b*-F8465 (not included in ISOGG 2019), downstream of C2b1a1-CTS2657, is one of the founding paternal lineages of Mongolic-speaking populations [105]. However, C2b1b-F845 and C2b1a2-F3880 were identified at relatively high frequencies in Han Chinese individuals, and C2b1b-F845 was also found in TB-speaking populations [99]. In general, owing to the cultural practices of nomadic groups, the distribution of haplogroup C2-M231 and its sublineages in northern East Asia are intricate, but we can still find some special haplogroups that are either the founding paternal lineages of distinct ethnolinguistic populations or have a region-specific distribution following the improvement of resolution. Second, O2-M122 represented a certain proportion of the Altaic-speaking populations in East Asia, which might have been caused by interactions with Han Chinese people (Fig. 3B). Third, N-M231 was also found in the Altaic-speaking population of East Asia (Fig. 3B). However, due to the lack of research on the downstream region of N-M231 in East Asia, we can discover from previous studies that the haplogroups in Altaic-speaking populations of East Asia are influenced by ancient Mongolian Plateau populations and are mainly allocated to N1a-F1260, which is consistent with the occurrence of N1a-F1260 in ancient northern East Asians [46,81,106]. Finally, Turkic-speaking populations, which are mainly located in northwestern East Asia, play an essential role in the interaction between West and East Eurasian populations, leading to an intricate composition of paternal lineages such as R-M207, Q-M242, J2-M172, I-M170, G-M201, and H-M69 [107]. Furthermore, we also found this complexity of paternal lineages based on ancient DNA in northwest East Asia [53] (Fig. 4B–D). R1a1 and R1b1 are related to the expansion of steppe culture in the Bronze Age, such as in Afanasievo and Andronovo culture [53,108]. R1a1a-M17 occupies a significant proportion of northwestern East Asia, especially in Turkic-speaking populations, which is related to the expansion of Indo-European-speaking populations [55]. The presence of Q-M242 might be attributed to the migration of Siberian populations. J2-M172 was observed in a certain percentage of Uygurs, Tajiks, and Uzbeks, which were influenced by the eastward nomads from Central Asia [107]. Additionally, the sporadic distribution of I-M170, G-M201, and H-M69 in northwestern East Asia may be induced by the paternal gene pool of the migrants along the “Silk Road”. In conclusion, the Altaic-speaking populations in northern East Asia exhibit a complex paternal genetic history and diversity influenced by the factors of admixture and cultural practices, such as the expansion of the Mongol Empire and inflow from the central and eastern Steppe. Population migrations along the Silk Road enriched the paternal gene pool of northern East Asians but played a marginal role in paternal genetic scenarios.

### 3.3 Multiple founding lineages of southern East Asia and Southeast Asia

The emergence and development of rice agriculture in the Yangtze River strongly influence the genetic structure of populations in southern East Asia, which promotes the movement and interaction between different ethnolinguistic populations. There are five main linguistically different populations in southern East Asia, namely, HM-, AA-, TK-, AN-, and ST-speaking populations. Although some share the genetic constitution of paternal lineages, the dispersal frequency of lineages of southern East Asians differs. Pieces of evidence from historical documents suggest that three ancient populations originated in southern East Asia: the ancestors of the TK people and AN population in the lower Yangtze River, the ancestors of HM-speaking populations in the middle Yangtze River and the ancestors of the AA-speaking populations in the upper Yangtze River [109]. The HM-speaking populations mainly spread in the mountains of south-central and southwest East Asia. According to ancient DNA, Li et al. revealed that a high frequency of O2a2a1a2-M7 was found at the Daxi site in the middle of the Yangtze River [110]. The Daxi site is related to the ancestor of present-day HM people [111]. Furthermore, O2a2a1a2-M7 also occupies a relatively high proportion of present-day HM-speaking populations [48]. Combining these two points, we can infer that O2a2a1a2-M7 is one of the founding paternal lineages of HM-speaking populations. However, the divergence time of O2a2a1a2-M7 was more than ten kya. Xia et al. reported that the most recent common ancestor of O2a2-N5 (∼2330 BP), downstream of O2a2a1a2-M7, was close to the proto-HM (∼2500 BP) [112], further suggesting that O2a2-N5 was one of the founding paternal lineages of HM-speaking populations. O1a-M119 is considered one of the founding paternal lineages of TK-speaking and AN-speaking populations [19]. However, there are discrepancies in the distribution frequency of O1a-M119. For example, the haplogroup frequency of O1a-M119 decreases from east to west in TK-speaking populations [43,49,113,114]. O1a-M119 has a high frequency in the AN population in Taiwan but a low frequency in the AN population on Southeast Asian islands [115,116]. According to historical records, the TK people are descendants of the ancient Baiyue people. O1a-M119 was found only in the Liangzhu culture (3300–2100 BCE) and was thought to have been created by the ancient Baiyue people [110]. Taiwan's Dabenkeng culture (3000–2500 BCE) is supported by the ancestral creation of the AN population. According to previous studies, the AN population was considered to be derived from TK-speaking populations [51,117]. Furthermore, Sun et al. revealed that Neolithic communities in Southeast China were the ancestors of TK-speaking and AN-speaking populations and suggested the founding of paternal lineages under O1a-M119 in these two ethnic groups [19]. O1b1a1a-M95 is thought to be the founding paternal lineage of the AA-speaking populations. The high proportion of O1b1a1a-M95 in the AA-speaking populations of Southeast Asia is generally thought to have spread from southern China during the Neolithic period [[118], [119], [120]]. There are only three AA-speaking populations in East Asia: Blang, De'ang, and Wa. However, the frequency of haplogroup O1b1a1a-M95 in these three populations was relatively lower than that in AA-speaking populations in Southeast Asia, which might be influenced by the surrounding ethnolinguistic populations [113,121]. ST populations in southern East Asia are mixed with local indigenous ancestors and thus acquire paternal gene pools of indigenous plants, such as O1a-M119, O2a2a1a2-M7, and O1b1a1a-M95. Due to long-term spatial and temporal contact, the indigenous paternal lineages O1a-M119, O2a2a1a2-M7 and O1b1a1a-M95 are widespread in the ethnolinguistic populations of southern East Asia. In summary, the emergence of rice agriculture had a crucial effect on the gene flow between the ancestors of the southern indigenous populations. The southward migration of ST populations also impacted the gene pool in southern East Asia.

## 4 New advances on the Y chromosome in the massively parallel or long-read sequencing era

The properties of Y chromosomes, including haploid inheritance and sensitivity to genetic drift, make them an essential tool for tracing human origin and evolution [122]. In forensic practice, Y-SNPs and Y-STRs are the most common genetic markers, playing a significant role in complex pedigree searches and paternal biogeographical ancestry inference. Y-chromosome haplogroups, with geography-specific and population-specific distributions, can be used to infer paternal biogeographic ancestry, which can provide some clues for the crime scene when the autosome STR profiles of the victim or perpetrator are unmatched [18,123]. The growth of NGS and the development of variant calling algorithms and tools have led to an exponential increase in novel Y-SNPs [6]. We detected unique distribution patterns of Y chromosome haplogroups in different geographic regions and ethnolinguistically diverse populations. However, the reliability of the PBGAI in forensic practice needs a comprehensive population reference database for verification [18,82,123]. The Y chromosome Haplotype Reference Database (YHRD) is one of the largest publicly available databases dedicated to collecting and providing information on Y chromosome Y-STR and Y-SNP haplotypes [124]. However, the delay in updating the YHRD database, disparities in data coverage across regions and populations, and the limited coverage of Y-STR and Y-SNP loci provide limited genetic information for forensic applications. Moreover, large-scale population-specific genomic sources in East Asia, such as the Westlake BioBank for Chinese (WBBC) [125] and the China Metabolic Analytics Project (ChinaMAP) [126], have ignored Y chromosome studies. In the future, high-quality population-specific genomic sources targeting the Y chromosome will shed light on population evolution and forensic applications.

### 4.1 The development of NGS-based Y chromosome panels

In the past two decades, forensic scientists have made significant progress in advancing the application of Y-SNPs in forensic practice. Based on investigations of the paternal gene pools of different populations, forensic scientists have developed several targeted NGS-based panels for forensic DNA investigation. The use of population-specific panels will increase the resolution of identifying terminal paternal haplogroups, enhancing the accuracy of forensic pedigree searches and biogeographical ancestry inference.

### 4.2 The identification of Y-SNP lineage information

Language division represents the separation of populations [127]. As mentioned above, the populations divided by language family present different founding paternal lineages, such as O–N5 in HM-speaking populations and C2a*-F5484 (not included in ISOGG 2019) in Tungusic-speaking populations. In the future, with the increase in high-coverage and high-depth sequencing data, revealing the founding paternal lineage of each ethnolinguistic population, with resolution from the language family to the language branch, is a common challenge for molecular anthropology and forensics.

### 4.3 The correlation between surname and Y chromosome haplogroup

Males within a pedigree usually share identical patrilineal surnames and similar Y chromosomes, which can reduce the scope of investigations in forensic practice by analyzing the Y chromosome to predict the surname of the perpetrator [28,128,129]. In China, Hereditary surnames can be traced back approximately 4000 years [130,131] and encompass the properties of long-term conservation, stability, and continuity owing to particular cultural traditions [132]. Previous studies have mainly concentrated on the correlation between Y-STRs and surnames [[133], [134], [135]], which more or less overlook the characteristics of Y-SNPs, decreasing the likelihood of false positives [[136], [137], [138]]. Due to the high mutation rate of Y-STRs and the limited number of loci, unrelated individuals may occasionally exhibit the same Y-STR haplotype. In contrast, the low mutation rate of Y-SNPs enables the determination of whether two individuals share a common ancestor, thereby reducing the occurrence of false positives. In addition, finding typical lineages based on geographic subregions within large pedigrees that share identical surnames, such as O-FGC66168, which is related to the surname Ye in China, will be helpful in forensic practice [139].

### 4.4 The advantage of long-read technology for the Y chromosome

Finally, the Telomere-to-Telomere (T2T) consortium recently filled the gaps in the Y chromosome, which accounts for approximately five-sixths of the genome but is only concentrated on one Y chromosome allocated to J1a-L816 [140]. The complete human Y chromosome elucidates numerous previously undisclosed genetic variations, revealing an abundance of repetitive sequences within the Y chromosome. Hallast et al. assembled 43 diverse Y chromosomes via long-read sequencing, which revealed nucleotide-level population variations in the Y chromosome and underscored the necessity of genome diversity of the Y chromosome [141]. Moreover, previously unresolved regions, including the heterochromatin region, also encompass the phylogenetic context, providing more insight into human evolution. The telomere-to-telomere assembly of the Y chromosome in East Asia, exemplified by CN1 and T2T-YAO, has additionally delineated variations in length and sequencing context compared to T2T-Y [142,143]. The utilization of sequence-resolved Y-chromosomes, previously inaccessible due to technical limitations, presents both an opportunity and a challenge in future forensic applications, such as the practical application of newly identified Y-STRs and Y-SNPs in the previously unresolved region of the Y-chromosome and the accuracy of calling Y-STRs from sequencing data. While the telomere-to-telomere assembly of the Y chromosome provides a novel reference benchmark for multiple disciplines, the graph-based pangenome reference of the Y chromosome will shed light on the identification of genetic variation. However, the recently published pangenome reference does not include the Y chromosome.

## 5 Conclusion

In this review, we presented the patterns of the diversity of Y chromosome haplogroups and the basic landscape of the paternal genetic history in East Asia from the perspectives of ancient and present-day paternal lineages. We also summarize the advances in sequencing technologies, the benefits of human genomic projects, and the corresponding applications of Y chromosomal genetic markers in forensic practice (Fig. 1). As mentioned above, East Asians not only are diverse in terms of linguistic, ethnic, and cultural background but are also rich in genetic diversity. We presented the distribution of major East Asian-related founding lineages based on the genetic variations of spatiotemporally different populations, mainly focused on the O, C, D, and N lineages in East Asians. The identified patterns of the composition of major founding lineages in modern and ancient people suggested that complex population migration and admixture contributed to the modern patterns of language- or geography-related dominant lineages. Advances in forensic high-resolution Y-chromosome systems have shown that findings on human evolution could provide a wealth of lineage-informative Y-SNPs for inferring paternal biogeographic ancestry in forensic science when no information about victims and perpetrators is obtained. We also emphasized the importance of including more ethnolinguistically diverse unrepresented populations in future genomic projects, and we believe that the T2T-based Y chromosome should help reconstruct the full landscape of the paternal evolutionary history of East Asians.

## Ethics approval and consent to participate

Not applicable.

## Consent for publication

Not applicable.

## Data availability

The allele frequency data derived from human samples have been submitted to the supplementary materials.

## CRediT authorship contribution statement

**Zhiyong Wang:** Writing – review & editing, Writing – original draft, Conceptualization. **Mengge Wang:** Writing – review & editing, Writing – original draft, Conceptualization. **Liping Hu:** Visualization, Data curation, Conceptualization. **Guanglin He:** Writing – review & editing. **Shengjie Nie:** Writing – review & editing, Conceptualization.

## Declaration of competing interest

The authors declare that they have no competing interest.
